# 

**Published:** 1842-10-29

**Authors:** 


**CONTENTS OF No. 44.**

Miller's case of Retention of the Fœtus in Utero, three years and ten months after the full term of Utero-Gestation, - - 689
BIBLIOGRAPHICAL NOTICE: The History, Diagnosis, and Treatment of Typhoid and Typhus Fever; with an Essay on the Diagnosis of Bilious Remittent and of Yellow Fever. By Elisha Bartlett, M. D., - - - - - - - - - 693
ANALECTA: Cases of Acute Hydrocephalus treated by the Iodide of Potassium, - - - - - - - - - 695
Jameson's Extraordinary case of Twins, - - - - 697
Hydatids in the Bones of the Pelvis, - - - - 698
Aneurism of the External Iliac Artery, - - - - 699
Influence of Tobacco on Phthisis, - - - - 702
Tincture of Creasote, - - - - - - - - 702
Deafness cured by the Endermic use of Morphia, - - 703
Fracture of the fore-arm, - - - - - - - 703
Removal of Discolouration of the Skin from Nitrate of Silver, 703
Chapped Nipples, - - - - - - - - - 704
Table of two hundred and twenty cases of Fracture, - - 704

